# A Rare Case Report of a Large Dentigerous Cyst in the Maxillary Sinus Associated With an Ectopic Maxillary Third Molar

**DOI:** 10.1155/crid/2436615

**Published:** 2025-07-03

**Authors:** Marika Ramishvili, Leila Atskvereli, Giorgi Menabde, Marika Zurmukhtashvili, Sopio Samkharadze, Giorgi Dugashvili, Luc Marks

**Affiliations:** ^1^Department of Maxillofacial Surgery, Faculty of Medicine, Ivane Javakhishvili Tbilisi State University, Tbilisi, Georgia; ^2^Department of Oral Surgery and Implantology, Tbilisi State Medical University, Tbilisi, Georgia; ^3^S. Khechinashvili University Hospital, Tbilisi, Georgia; ^4^Faculty of Dentistry, European University, Tbilisi, Georgia; ^5^School of Medicine, Grigol Robakidze University, Tbilisi, Georgia; ^6^Faculty of Medicine & Health Sciences, University of Antwerp, Antwerp, Belgium; ^7^Special Care in Dentistry, Department of Cranio-Maxillofacial Surgery, Antwerp University Hospital, Edegem, Belgium

**Keywords:** Caldwell–Luc approach, cystic lesion, intrasinusal position of the molar, maxillary sinusitis, sinus opacification

## Abstract

Ectopic eruption of permanent molars is an uncommon developmental anomaly characterized by abnormal tooth positioning, which can lead to significant complications. In rare instances, ectopic molars may be associated with dentigerous cysts, particularly within the maxillary sinus, posing challenges for diagnosis and management. This report discusses a rare case of a 58-year-old male who presented with chronic right maxillary sinusitis, intermittent facial pain, and purulent nasal and oral discharge. Radiological evaluation, including cone beam computed tomography (CBCT), revealed a completely opacified right maxillary sinus containing an ectopic maxillary molar. Additionally, a large cystic lesion consistent with a dentigerous cyst was found, occupying the entire sinus cavity. Surgical management was performed using the Caldwell–Luc approach under general anesthesia. This involved creating a bone window in the anterior maxillary wall to facilitate the removal of the ectopic tooth and the associated cystic lesion. Histopathological examination confirmed the presence of a dentigerous cyst exhibiting chronic inflammatory infiltration and fibrosis. Ectopic molars in the maxillary sinus are often asymptomatic but can present with recurrent sinusitis, pain, and oroantral communication. The existence of a large dentigerous cyst heightens the risk of complications and may obscure radiological interpretation due to sinus opacification. This case highlights the necessity of comprehensive imaging and early surgical intervention to prevent long-term complications. Awareness of such rare conditions can help clinicians in prompt diagnosis and appropriate management, ultimately preserving sinus function and minimizing further issues.

## 1. Introduction

Ectopic eruption refers to a developmental disturbance in which a tooth emerges along an abnormal trajectory, rather than following its typical eruption pathway. Developmental anomalies, dental trauma leading to tooth displacement, maxillary infections, crowding, genetic predisposition, and increased bone density can be regarded as etiological factors [1]. These factors can lead to the permanent molars erupting in proximity to the distal undercut of the second deciduous molar or first permanent molar, respectively, rather than emerging into the typical occlusal alignment [2].

In the 2018 study of Güven and colleagues, ectopic eruption of first permanent molars was observed in 2.65%. Among the affected cases, the majority of ectopic firs permanent molars were located in the maxilla, accounting for 57.5%, while 42.5% were found in the mandible [3]. Ectopic eruption of maxillary first permanent molars was more prevalent in males and at younger ages. Additionally, individuals with skeletal Class III malocclusion showed a higher likelihood of being diagnosed with this condition [4]. Impaction of mandibular second molars is more frequently observed than that of maxillary second molars [5, 6]. In terms of gender, there was demonstrated no difference in most of the studies for both ectopic first and second permanent molars. However, Güven and Aldowsari reported a lower incidence in females among Turkish and Saudi populations [3, 7].

The experts in various literature recognize this condition as a multifaceted pathological issue, intricately influenced by both general and local factors that can function independently or interactively. Key general factors include significant familial predisposition and genetic influences, underscoring the importance of understanding these underlying elements in addressing this dental concern [8]. Several significant local factors have been identified, particularly the imbalance between mandibular growth and the eruption of the first permanent molar. This crucial imbalance can result in a persistent mesial inclination or an abnormal eruption angle for the molar, ultimately causing it to become trapped beneath the distal bulge of the second primary molar. This phenomenon highlights the importance of understanding the interplay between growth patterns and dental eruption to prevent potential complications [9, 10].

Ectopic teeth have various locations. In the review by Ngoc et al., 33 patients were examined, and in comparison, to the position within the maxillary sinus, 16 cases (48.5%) were situated on the right side, while 17 cases (51.5%) were on the left side [11]. These occurrences are likely distributed across various regions of the sinus, including the antrum, floor, roof, orbital floor, and superomedial and anterosuperior aspects, as well as the posterior and anterolateral walls. In a recent study conducted by Masalha, 11 ectopic teeth within the maxillary sinus across 10 patients were identified [12]. These cases were distributed across various locations including medial and lateral walls, orbital floor, posterior wall, and maxillary sinus floor. In addition, one case reported ectopic molar to be identified at the junction between the orbital floor and medial wall [13]. Additionally, while the majority of reported cases of ectopic teeth in the maxillary sinuses were unilateral, bilateral occurrences have also been documented in the literature [13].

Diagnosing an ectopic tooth in the maxillary sinus is often a clear and manageable process when utilizing advanced radiological investigations. Techniques such as panoramic radiography (OPG), CT scans of the nose and paranasal sinuses, and cone beam computed tomography (CBCT) provide detailed insights. A definitive radiological indicator of an ectopic tooth is the identification of a tooth-like bony structure situated outside the typical dentate region, highlighting the effectiveness of these imaging methods in enhancing diagnostic accuracy [13].

Although ectopic teeth in the maxillary sinus can sometimes be asymptomatic, a majority of cases are associated with various clinical features such as headache, heaviness or pain over the cheek, facial pain, cheek swelling, dull pain in the region of the maxillary sinus, purulent nasal or postnasal discharge, hyposmia, recurrent facial swelling, foul odor, radiating pain to the eye and ear, chronic cough, and blurred vision [13–15]. In bilateral cases can occur bilateral facial pain and swelling [16, 17]. However, it should be mentioned that these symptoms can arise from a variety of conditions and are not solely indicative of ectopic third molars. It is essential to consider other potential causes for accurate diagnosis and treatment.

The presence and severity of these diverse symptoms, whether experienced individually or in combination, may depend on several factors, including the specific location of the ectopic tooth within the maxillary sinus, the duration of its presence, and whether any associated pathology such as infection, cysts, or tumors is present or not [13, 15]. In 1.44%, unerupted teeth are associated with dentigerous cysts [18]. In a 2024 study by Aldelaimi and colleagues in the evaluated cases of patients with dentigerous cysts, 85.5% were diagnosed as dentigerous cysts without associated anomalies, with 50% occurring in the mandible and 35.5% in the maxilla. The remaining 14.5% presented as mixed lesions involving other anomalies, such as odontomas. Among these, 13.1% were located in the mandible and 1.3% in the maxilla [19].

The treatment of a dentigerous cyst is typically straightforward and involves extraction of the associated tooth followed by thorough curettage of the surrounding soft tissue. However, due to the potential for extensive tissue destruction associated with this approach, more conservative alternatives—such as marsupialization, endoscopy, and cryotherapy—can be considered. Extensive lesions may be managed through marsupialization to reduce cyst size prior to definitive treatment [19, 20]. These methods, either alone or in combination, are aimed at preserving adjacent anatomical structures. The choice of technique depends on the extent of the lesion and patient-specific factors, requiring thorough evaluation to ensure optimal outcomes in terms of both safety and comfort.

We aimed to report the rare clinical case. During embryogenesis, influence of unknown etiologic factors caused the development of the follicle of Tooth #1.8 in the maxillary sinus. Our interest was increased due to the presence of a large dentigerous cyst originating from ectopic Tooth #1.8 in the maxillary sinus.

## 2. Case Presentation

Data collection was approved by the local ethics committee of the clinic. Written informed consent was obtained from the patient.

The patient, a 58-year-old male, came to S. Khechinashvili University Hospital with a diagnosis of exacerbated chronic sinusitis of the right maxillary sinus and ectopy of the right maxillary molar in the maxillary sinus.

**Figure 1:**
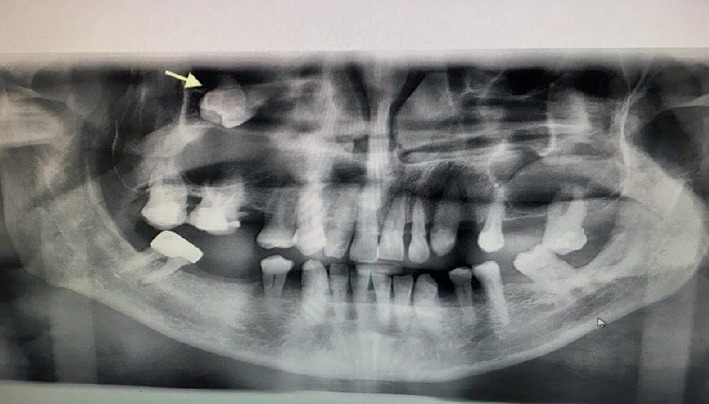
Preoperative dental panoramic radiograph showing ectopic tooth (arrow) in the right maxillary sinus.

**Figure 2:**
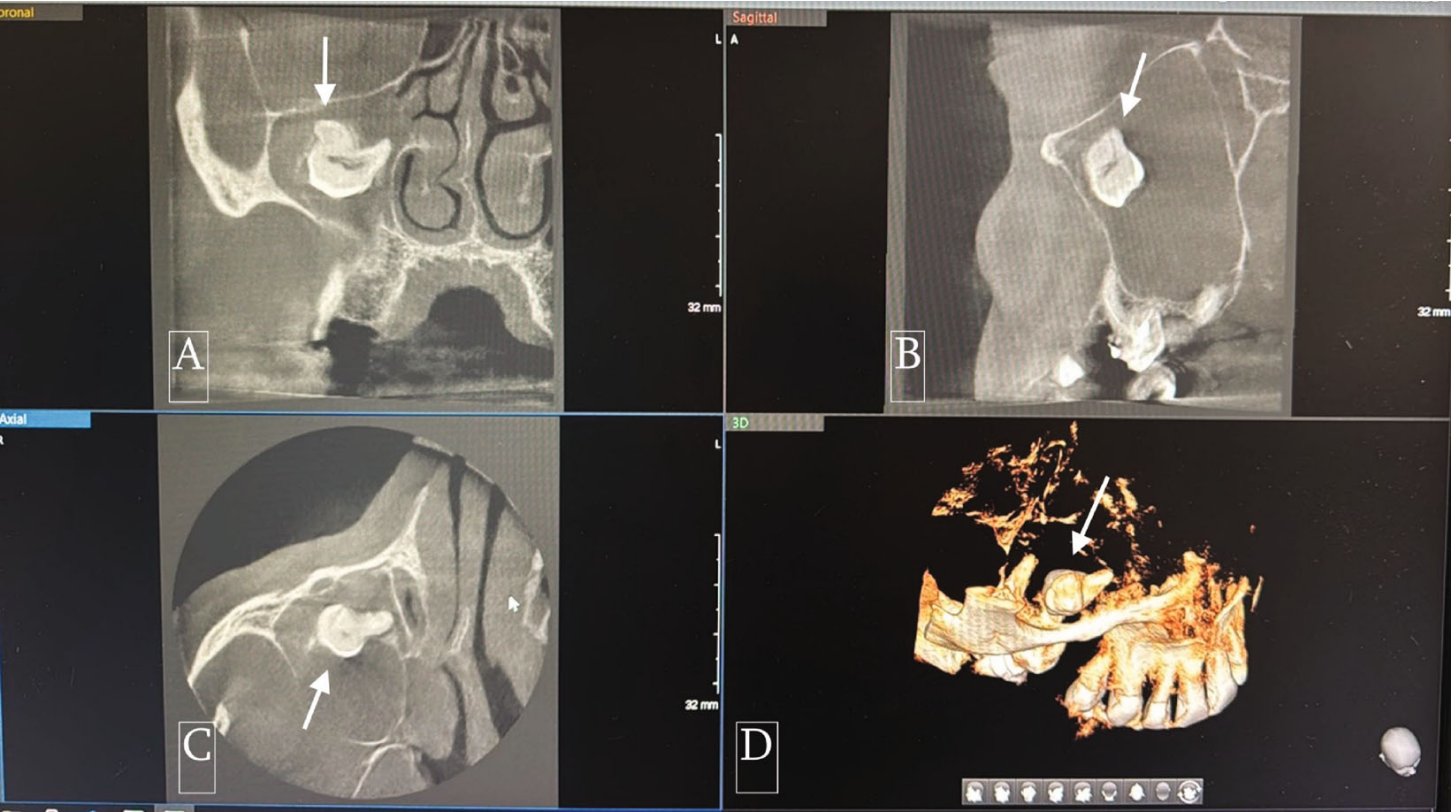
Preoperative cone beam computer tomography image showing ectopic tooth (white arrows) in the right maxillary sinus. (A) Coronal view. (B) Sagittal view. (C) Axial view. (D) 3D reconstruction.

**Figure 3:**
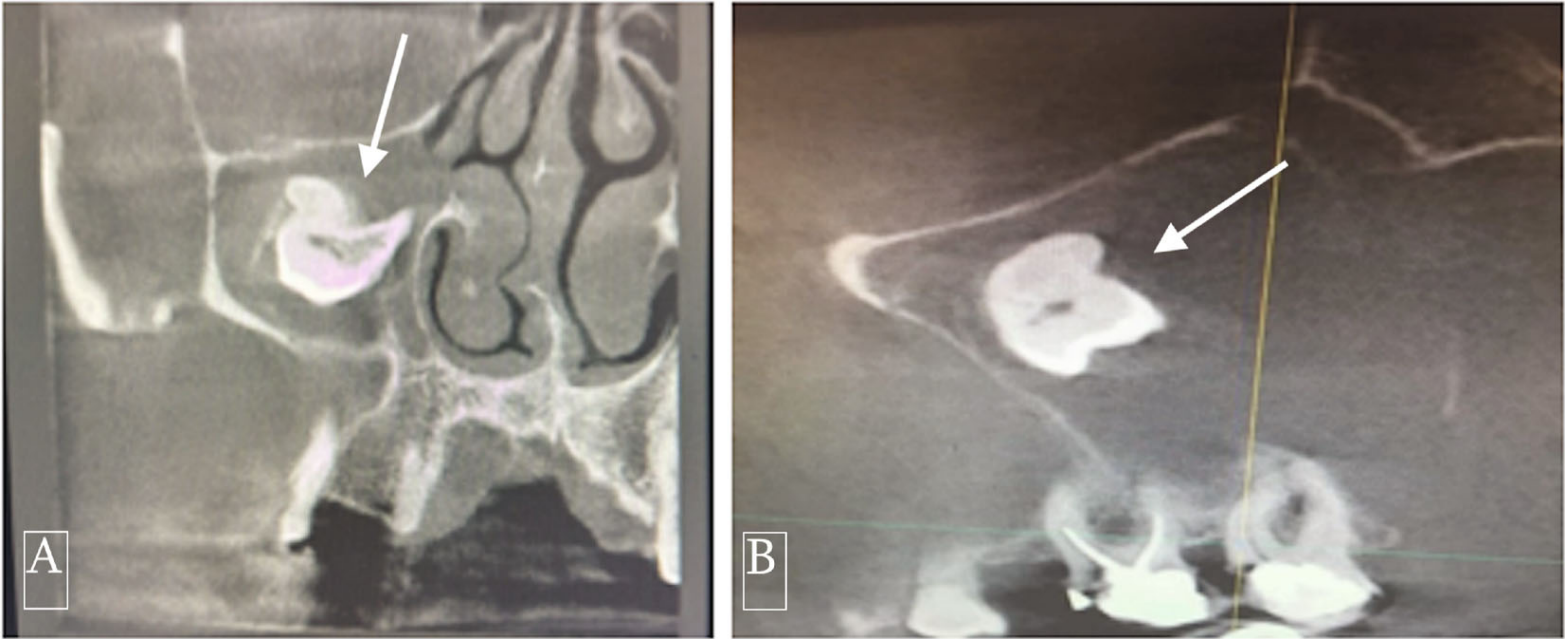
Preoperative cone beam computer tomography images (the best visualizations) showing ectopic tooth (white arrows) in the right maxillary sinus. (A) Coronal view. (B) Sagittal view.

**Figure 4:**
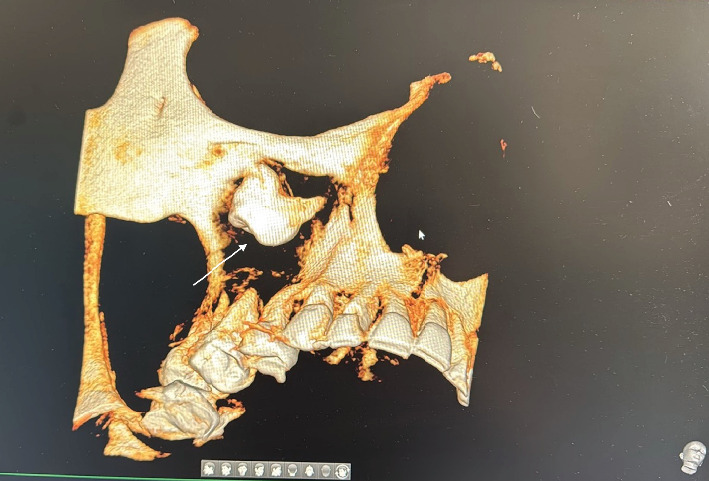
The best view of 3D reconstruction showing ectopic tooth (white arrow) in the right maxillary sinus.

**Figure 5:**
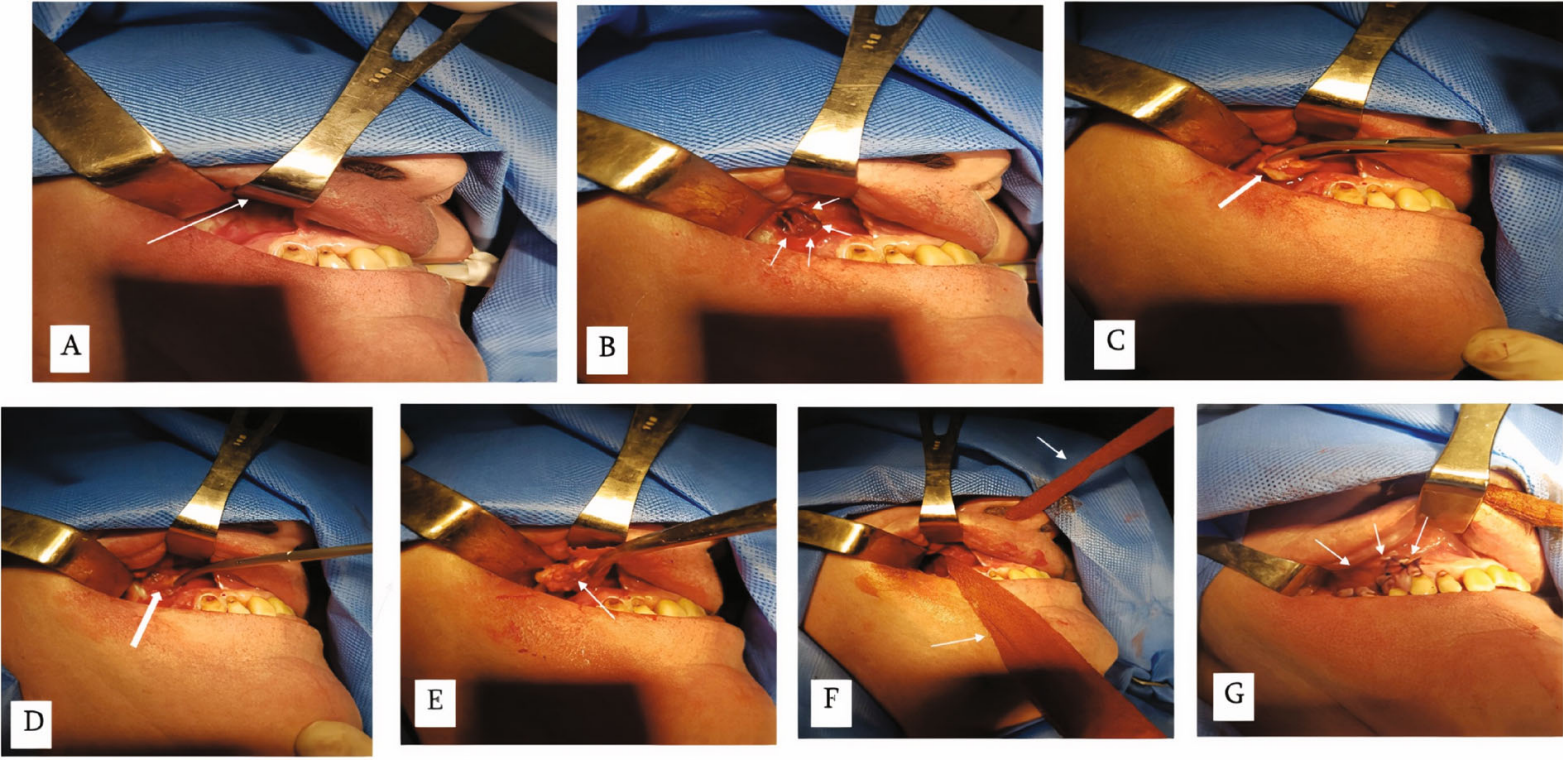
Intraoperative photographs. (A) Soft tissue flap is mobilized. (B) Bone window is formed (arrows). (C) Ectopic molar (arrow) removal. (D, E) Extirpation of the cyst wall (cyst—arrows). (F) Pledget in the nasal cavity (arrows). (G) Wound after suturing (arrows).

**Figure 6:**
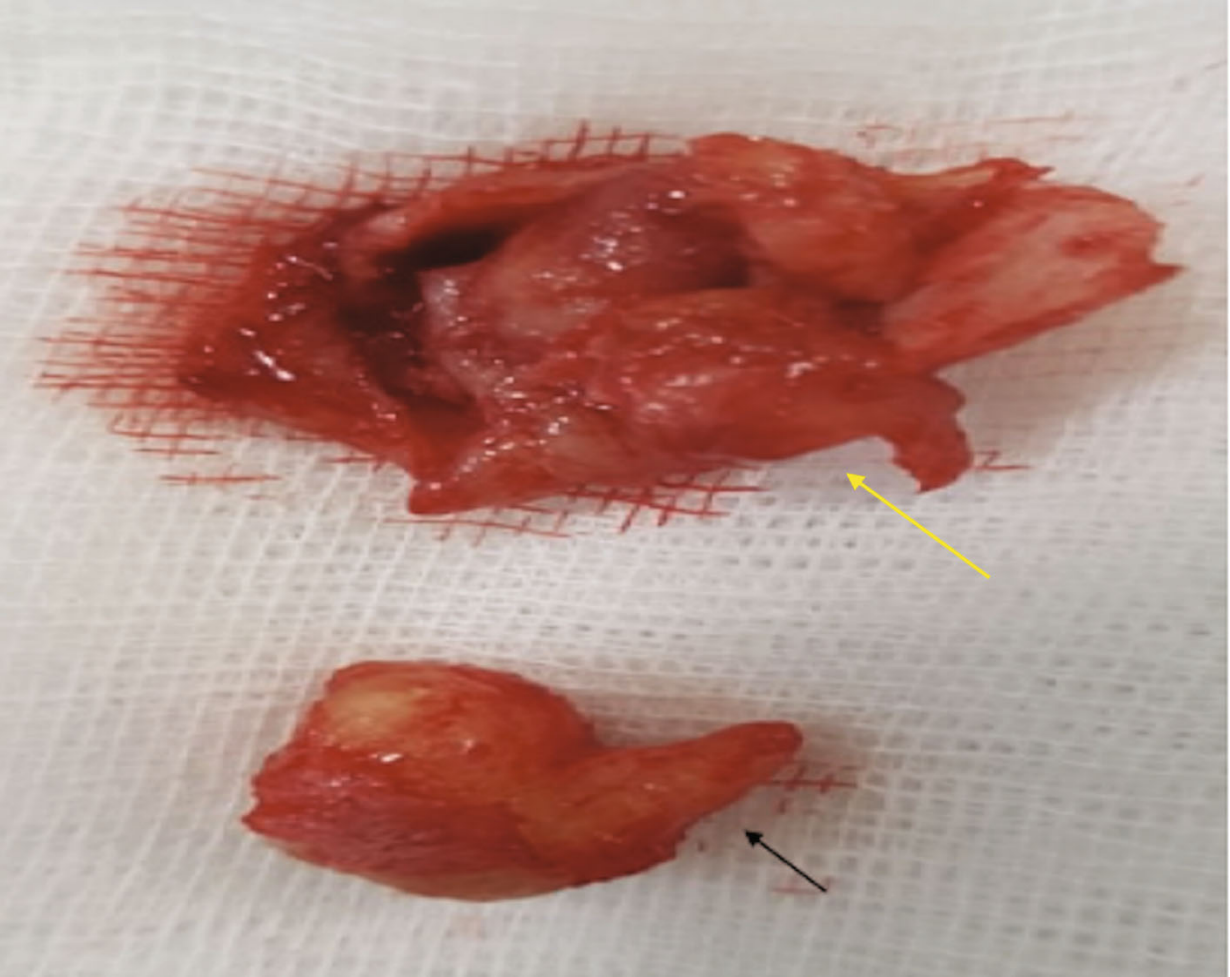
Extirpated ectopic molar (black arrow) and objects of inflamed mucosa of the sinus (yellow arrow).

The patient reported pain in the right maxilla in the past 2 years with periodic intermissions. A new phase of exacerbation has begun, with inflammatory infiltration of soft tissues adjacent to the right maxilla and purulent exudate from the nasal and oral cavity. The patient came to the dental clinic, where he was subjected to examinations. CBCT of facial bones was done (PaX-i3D, Vatech, Korea, with a scan time of 18 s at 94 kVp and 8.1 Ma, dose-area product (DAP) of 601.41 mGy·cm^2^); digital data were analyzed by software. CBCT examination revealed homogeneous total opacification and ectopic molar in the right maxillary sinus. In addition, root apices of Tooth #1.7 were penetrated in the maxillary sinus, and inflammatory destruction of bone in the region of tuber maxillae was present (Figures 1, 2, 3, and 4). Intraoral examination revealed anodontia of Tooth #1.5 and gangrenous Tooth #1.7. The patient reported that Tooth #1.5 had been extracted 4 years before. At the projection of Tooth #1.7 from the vestibular side, an oroantral fistula was present with periodic discharge of pus. Deep probing revealed communication of the oroantral fistula with the maxillary sinus. According to these findings, we concluded that chronic sinusitis with intermittent exacerbations, which was the reason the patient came to the clinic, was associated with gangrenous Tooth #1.7. The ectopic molar and associated dentigerous cyst located in the maxillary sinus were not diagnosed previously and were incidental findings: the ectopic tooth was diagnosed during the preoperative examination; the dentigerous cyst was diagnosed intraoperatively (Figures 5 and 6). We suppose that, in the preoperative period, the dentigerous cyst was not diagnosed because the maxillary sinus was full of the dentigerous cyst. Accordingly, the cyst wall was tightly lined at the walls of the maxillary sinus. During the radiographic examination, the cyst wall was closely adherent to the maxillary sinus walls, and the superimposition of anatomical structures prevented the identification of the cyst.

In the preoperative period, the patient was subjected to all necessary examinations (blood tests, electrocardiography, and abdominal ultrasound examination). In addition, to prevent postoperative complications, the irrigation procedures of the maxillary sinus with an antiseptic solution were performed (five procedures).

Surgical intervention was performed under general anesthesia (endotracheal narcosis). On the right side, in the region of the alveolar ridge from canine to tuber maxillae, a Caldwell–Luc atypical incision was done. A soft tissue flap was mobilized (Figure 5A). Maxillary tuberosity and canine fossa were uncovered. The maxillary sinus was opened with bur, bone mass in size of 2.0 × 2.0 cm^2^ was removed, and the bone window was formed (Figure 5B). In the maxillary sinus, a small amount of purulent exudate was revealed and drained after the sinus was opened. Besides pus, a large dentigerous cyst has been visualized. The walls of the cyst filled the entire sinus. A cyst was developed around the maxillary ectopic molar (Ectopy Type III). In the region of the tuber, signs of inflammatory osteoporosis were present, with bone destruction in the periapical region of Tooth #1.7. In this area was an opened oroantral fistula with purulent discharge, which communicated maxillary sinus to the oral cavity. Purulent exudate has been fully drained from the maxillary sinus. To extirpate the cyst together with an ectopic tooth would be necessary to widen the bone defect in the anterior wall of the sinus and consequently increase the traumatization impact of intervention. We decided to remove the ectopic tooth and cyst separately. In the first step, content of the cyst was aspirated, after the wall of the cyst had been cut and the ectopic molar had been removed (Figure 5C). At the final stage, the wall of the cyst was extirpated as one object (Figure 5D,E). Tooth 1.7 was extracted. The damaged osteoporotic bone area of the tuber has been removed within healthy tissue margins. The purulent oroantral fistula has been also surgically removed. The sinus has been irrigated with an antiseptic solution; soft tissue flap was mobilized to cover the oroantral communication. The maxillary sinus has been filled with pledges with an antiseptic solution. One end of the pledget has been guided out into the lower nasal meatus (Figure 5F). The wound has been sutured in the projection of the tuber with the closing of oroantral fistula, with atraumatic sutures (2/0) and simple interrupted technique with knots (Figure 5G). Antimicrobial therapy was performed according to microbiological evaluation: Rocephin 1.0 g every 12 h for 3 days in combination with metronidazole 0.5 g every 8 h intravenously. Twenty milligrams of H1-blocker chloropyramine hydrochloride (Suprastin) was given intramuscularly every 12 h. The patient stayed at the hospital for 3 days On Day 3 after surgery, the pledget was removed from the maxillary sinus. The patient's overall condition was satisfying and after that treatment continued on an outpatient care basis. The wound has healed with primary intention. Sutures have been removed on Day 8 after surgery.

Specimen—a wall of the cyst as one block with three objects of inflamed mucosa of the sinus (Figure 6)—has been subjected to morphological evaluation in the research center of pathology. Postoperative clinical diagnosis has been stated as follows: right chronic exacerbated maxillary sinusitis, large dentigerous cyst of the maxillary bone, originated from ectopic molar in the maxillary sinus.

Morphological evaluation was done under a light microscope. Specimen was stained with hematoxylin–eosin and picrofuxin with Van Gieson's stain methodology. The morphological assay revealed cholesterol crystals and minimal plasmocyte and lymphocyte infiltration. In the outer layer of the cyst wall, fibrosis was present whereas in the inner layer stratified, squamous epithelial cells were present. No atypical cells have been found. In the soft tissue, fragments of maxillary sinus mucosa edema of subepithelial stroma, seromucinal glands without atypical cells, moderate lympho- and plasmocyte, eosinophilic, and neutrophilic infiltration sites have been revealed. Morphological diagnosis stated dentigerous cyst of the maxillary sinus, with sites of fibrosis and nonspecific inflammatory infiltration in the walls, chronic exacerbated nonspecific maxillary sinusitis. Morphological diagnosis was in full accordance with clinical diagnosis.

## 3. Discussion

Ectopic eruption of permanent molars into the maxillary sinus is a dental anomaly that can present significant diagnostic and therapeutic challenges. The displacement of these teeth may occur due to various factors, including abnormal tooth germ migration, trauma, genetic predisposition, or pathological conditions such as odontogenic cysts and tumors. In our case, the ectopic maxillary molar was associated with a large dentigerous cyst, which had expanded within the sinus cavity, leading to chronic sinusitis symptoms.

The clinical presentation of ectopic molars within the maxillary sinus varies. While some cases remain asymptomatic and are incidentally discovered on radiographic imaging, others present with symptoms such as headache, recurrent facial swelling, dull pain in the region of the maxillary sinus, and purulent nasal or postnasal discharge. Our patient exhibited chronic sinusitis with periodic pain and recurrent nasal and oral discharge, which warranted further investigation. The use of CBCT was instrumental in identifying the ectopic tooth and the associated lesion, providing valuable insights into the extent of sinus involvement.

In rare cases (5%–6% of all dentigerous cysts), ectopic teeth are associated with dentigerous cysts [21, 22]. Dentigerous cysts are the predominant developmental cysts found in the jaws, ranking second only to radicular cysts among odontogenic cyst types. Also referred to as dentigerous cysts, they arise due to fluid buildup between the reduced enamel epithelium and the enamel surface of a fully formed tooth. These cysts originate from the detachment of the follicle surrounding the crown of an unerupted tooth [23]. When these cysts enlarge, they can cause significant bone resorption, sinus expansion, and secondary infection. In our case, the cyst occupied a substantial portion of the maxillary sinus, necessitating surgical intervention.

Surgical options for managing ectopic teeth generally fall into two categories: internal and external approaches. An internal approach may involve the Caldwell–Luc operation and/or endoscopy-assisted removal. On the other hand, the external approach typically consists of a midfacial degloving procedure. The decision regarding the surgical method is critically influenced by the position of the ectopic tooth and any accompanying conditions, such as sinusitis or dentigerous cysts. Understanding these factors is essential to ensure effective treatment and optimal patient outcomes [13]. The final aesthetic and cosmetic outcomes are vital factors to consider when choosing an approach, as they significantly impact the overall success and satisfaction of the results [14, 24]. In our case, the Caldwell–Luc approach was chosen because of the extensive sinus involvement and the need for complete cyst and tooth removal. This technique provided adequate access to the sinus while minimizing the risk of complications such as oroantral fistula formation. Histopathological analysis confirmed the diagnosis of a dentigerous cyst with inflammatory changes, highlighting the importance of early surgical management. Delayed intervention in such cases can lead to complications, including sinus obliteration, secondary infections, or even more severe maxillary bone resorption. The surgical outcome in our patient was favorable, with resolution of symptoms and no recurrence observed during follow-up.

This case underscores the importance of thorough radiological assessment and prompt intervention in patients presenting with maxillary sinus pathology of unknown origin. In our case, adherence of the cyst walls to the sinus walls and superimposition of anatomical structures prevented the identification of the cyst preoperatively. Given these challenges, it is essential to analyze maxillary sinus CBCT scans meticulously across multiple planes, including axial, coronal, and sagittal sections. It is particularly important to pay close attention to subtle changes in the sinus contour, bone remodeling, or the displacement of adjacent structures, as these may indicate the presence of a lesion that could be obscured by overlapping anatomy. Additionally, the possibility of adherent cysts should be considered, especially when the sinus appears opacified or when clinical symptoms are persistent and unexplained. This should trigger a more detailed radiological review. A comprehensive evaluation with a heightened level of suspicion can help prevent missed diagnoses and ensure timely and appropriate surgical planning. As demonstrated in this case, even advanced imaging modalities like CBCT may not reveal certain lesions unless they are interpreted with particular attention to these potentially hidden presentations.

Clinicians should consider ectopic teeth as a potential etiology in cases of chronic sinusitis, particularly when standard treatments fail to provide relief. Advanced imaging techniques such as CBCT can aid in diagnosis, while surgical approaches should be tailored to the individual case to ensure optimal outcomes. Future research on the etiology and management of ectopic molars in the maxillary sinus will contribute to a better understanding and improved treatment strategies for such rare cases.

We revealed in our clinical practice the rare case of tooth ectopy: formation of maxillary molar in the maxillary sinus. In addition, we as clinicians were interested in the fact that, intraoperatively, a large dentigerous cyst originated from an ectopic tooth, which filled the maxillary sinus, and which was missed in diagnosis before surgical intervention on the CBCT.

## Data Availability

The data supporting the findings of this study are available from the corresponding author upon reasonable request. All relevant data have been anonymized to protect participant confidentiality and comply with ethical standards.

## References

[1] Sharma S., Chauhan J. S. (2019). Bilateral Ectopic Third Molars in Maxillary Sinus Associated With Dentigerous Cyst-a Rare Case Report. *International Journal of Surgery Case Reports*.

[2] Alfuriji S., Alamro H., Kentab J. (2023). Ectopic Permanent Molars: A Review. *Dentistry Journal*.

[3] Güven Y. (2018). Prevalence of Ectopic Eruption of First Permanent Molars in a Turkish Population. *European Oral Research*.

[4] Zhang K., Zhang Y., Ma Y. (2024). Ectopic Eruption of Maxillary First Permanent Molars: Risk Factors and Association With Alveolar and Maxillary Characteristics on Children. *Journal of Dental Sciences*.

[5] Turley P. K. (2020). The Management of Mesially Inclined/Impacted Mandibular Permanent Second Molars. *Journal of the World Federation of Orthodontists*.

[6] la Monaca G., Cristalli M. P., Pranno N., Galluccio G., Annibali S., Pippi R. (2019). First and Second Permanent Molars With Failed or Delayed Eruption: Clinical and Statistical Analyses. *American Journal of Orthodontics and Dentofacial Orthopedics*.

[7] Aldowsari M. K., Alsaidan M., Alaqil M. (2021). Ectopic Eruption of First Permanent Molars for Pediatric Patients Attended King Saud University, Riyadh, Saudi Arabia: A Radiographic Study. *Clinical, Cosmetic and Investigational Dentistry*.

[8] Helm A., Martín-Vacas A., Molinero-Mourelle P., Caleya A., Gallardo N., Mourelle-Martínez M. (2021). Ectopic Eruption of Maxillary First Permanent Molars: Preliminary Results of Prevalence and Dentoskeletal Characteristics in Spanish Paediatric Population. *Children*.

[9] Mucedero M., Rozzi M., Cardoni G., Ricchiuti M. R., Cozza P. (2015). Dentoskeletal Features in Individuals With Ectopic Eruption of the Permanent Maxillary First Molar. *Korean Journal of Orthodontics*.

[10] Aldowsari M. K., Sulimany A. M., Alkhathlan A. (2025). Prevalence of Dental Anomalies in Pediatric Patients at King Saud University Dental Hospital, Riyadh, Saudi Arabia-A Radiographic Analysis. *Children*.

[11] Ngoc V. T. N., Anh L. Q., Duc N. M., Dinh T. C., Dinh T. C. (2019). Cone Beam Computed Tomography Application in Finding Ectopic Tooth: A Systemic Analysis and a Case Report. *Open Access Macedonian Journal of Medical Sciences*.

[12] Masalha M., Schneider S., Kassem F. (2021). Endoscopic Treatment of Ectopic Teeth in the Maxillary Sinus. *Journal of Clinical and Experimental Dentistry*.

[13] Al-Ani R. M., Aldelaimi T. N., Khalil A. A. (2022). Ectopic Upper Third Molar in Maxillary Sinus: A Case Report and Literature Review. *Indian Journal of Otolaryngology and Head & Neck Surgery*.

[14] Ramanojam S., Halli R., Hebbale M., Bhardwaj S. (2013). Ectopic Tooth in Maxillary Sinus: Case Series. *Annals of Maxillofacial Surgery*.

[15] Lai Y.-T. A., Luk Y. S., Fung K.-H. (2013). Anomalous Morphology of an Ectopic Tooth in the Maxillary Sinus on Three-Dimensional Computed Tomography Images. *Journal of Radiology Case Reports*.

[16] Elmorsy K., Elsayed L. K., El Khateeb S. M. (2020). Case Report: Ectopic Third Molar in the Maxillary Sinus With Infected Dentigerous Cyst Assessed by Cone Beam CT. *F1000Research*.

[17] Demirtas N., Kazancioglu H. O., Ezirganli S. (2014). Ectopic Tooth in the Maxillary Sinus Diagnosed With an Ophthalmic Complication. *Journal of Craniofacial Surgery*.

[18] Mourshed F., Aldelaimi A., Enezei H., Aldelaimi T. (2021). A Roentgenographic Study of Dentigerous Cysts: I. Incidence in a Population Sample. *Oral Surgery, Oral Medicine, Oral Pathology*.

[19] Aldelaimi A. A. K., Enezei H. H., Berum H. E. R., Abdulkaream S. M., Mohammed K. A., Aldelaimi T. N. (2024). Management of a Dentigerous Cyst; a Ten-Year Clinicopathological Study. *BMC Oral Health*.

[20] Ghandour L., Bahmad H. F., Bou-Assi S. (2018). Conservative Treatment of Dentigerous Cyst by Marsupialization in a Young Female Patient: A Case Report and Review of the Literature. *Case Reports in Dentistry*.

[21] Al-Ani R. M., Aldelaimi T. N., Khalil A. A., Abdulkareem S. M. (2024). Ectopic Upper Third Molar Embedded in a Dentigerous Cyst of the Maxillary Sinus: A Case Report and Literature Review. *Egyptian Journal of Otolaryngology*.

[22] Shah K. M., Karagir A., Adaki S., Pattanshetti C. (2013). Dentigerous Cyst Associated With an Impacted Anterior Maxillary Supernumerary Tooth. *Case Reports*.

[23] Neville B. W., Damm D. D., Allen C. M., Chi A. C. (2015). *Oral and Maxillofacial Pathology*.

[24] Lombroni L. G., Farronato G., Santamaria G., Lombroni D. M., Gatti P., Capelli M. (2018). Ectopic Teeth in the Maxillary Sinus: A Case Report and Literature Review. *Indian Journal of Dental Research*.

